# Supplementary material for: Age-dependent structural reorganization of utricular ribbon synapses
Source: Front Cell Dev Biol. 2023 Aug 10;11:1178992. doi: 10.3389/fcell.2023.1178992 (PMC10447907; doi:10.3389/fcell.2023.1178992)
Supplement: Supplementary file 1 [file Presentation1.pdf]

Movie 1-3: Electron tomography with 3D segmentation of ribbon cluster and single attached ribbons.
Tomograms and their corresponding 3D models of mature (3 months) type I/II VHCs depicting several disc-shaped floating ribbons in type I VHCs and typical single attached ribbons in type II VHCs. The different SV pools, membrane-proximal (MP)-SVs and ribbon-associated (RA)-SVs, are defined as displayed in Fig. S1A.
Red: ribbon, magenta: presynaptic density, dark blue: postsynaptic density, light blue: HC membrane, yellow: SVs, orange: MP-SVs, green: RA-SVs, brown: SV not associated with the ribbon but part of the cluster “cloud”. Scale bars: 200 nm.

Movie 1: https://figshare.com/articles/media/Movie_1/23723856
Movie 2: https://figshare.com/articles/media/Movie_2/23723871
Movie 3: https://figshare.com/articles/media/Movie_3/23723880

Movie 4 and 5: FIB-SEM visualizations of type I VHCs with 3D segmentation of single ribbons and ribbon clusters.
Movies scanning through the representative FIB-SEM z-stacks of P15 (Movie 4) and 8-month-old (Movie 5) type I VHCs, showing 3D reconstruction of the VHC contours (transparent gray), part of the nuclei (dark gray), synaptic ribbons (red) and their corresponding SVs (yellow). Movie 4 displays two neighboring P15 type I VHCs likely from the striola that are enclosed by one complex calyx.

Movie 4: https://figshare.com/articles/media/Movie_4/23723919
Movie 5: https://figshare.com/articles/media/Movie_5/23723919

Movie 6 and 7: FIB-SEM visualizations of the basolateral compartment of type II VHCs with corresponding 3D segmentations.
Movies scanning through the FIB-SEM z-stacks of P15 (Movie 6) and 8-month-old (Movie 7) type II VHCs. The corresponding 3D models depict VHC contours (transparent gray), part of the nuclei (dark gray), innervating nerve fibers (blue), mainly single attached ribbon synapses (red) and their corresponding SVs (yellow). Additionally, some afferent fibers possess multiple ribbons per bouton (Movie 6).

Movie 6: https://figshare.com/articles/media/Movie_6/23723937
Movie 7: https://figshare.com/articles/media/Movie_7/23723937

Movie 8: Ribbon cluster formation of a type I VHC.
Overview of a partial FIB-SEM z-stack showing a typical ribbon cluster formation at higher magnification of an 8-month-old type I VHC. Several floating ribbons can be observed in close proximity to the VHC membrane exhibiting no membrane attachment.

Movie 8: https://figshare.com/articles/media/Movie_8/23723667

Movie 9: Filamentous network at the nucleus level can be traced in a partial FIB-SEM z-stack of an 8-month-old type I VHC.
Movie displays a large microfilament network (highlighted with arrowheads) proximal to a ribbon cluster (highlighted by a red circle).

Movie 9: https://figshare.com/articles/media/Movie_9/23723943

Movie 10 and 11: 3D visualization of mitochondria from FIB-SEM z-stacks of P15 and 8-month-old type I and type II VHCs.
Movies show 3D reconstructions of VHC contours (transparent gray), part of the nuclei (dark gray) and mitochondria (randomly colored).

Movie 10 (P15):
https://figshare.com/articles/media/Movie_10_and_11/23723952?file=41652546
Movie 11 (8 months):
https://figshare.com/articles/media/Movie_10_and_11/23723952?file=41652549
