# Supplementary material for: Age-dependent structural reorganization of utricular ribbon synapses
Source: Front Cell Dev Biol. 2023 Aug 10;11:1178992. doi: 10.3389/fcell.2023.1178992 (PMC10447907; doi:10.3389/fcell.2023.1178992)
Supplement: Supplementary file 2 [file DataSheet1.DOCX]

**Supplementary information**


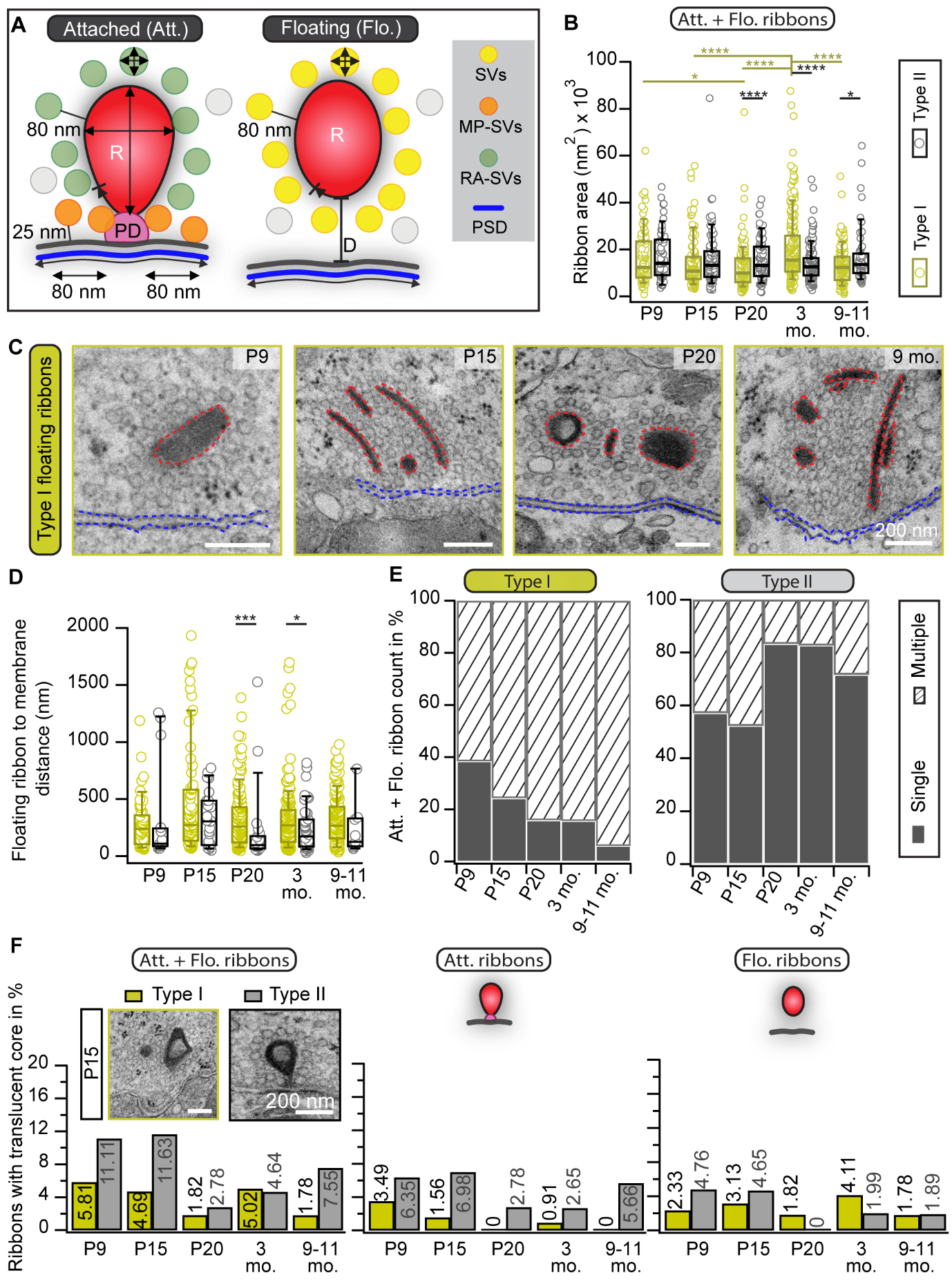


**Fig. S1: With advancing age the number of multiple ribbons rises in type I VHCs but reduces in type II VHCs.**

(A) Schematic representation of the applied quantification criteria. (B) Box plot of ribbon area measurements from both attached (Att.) as well as floating (Flo.) ribbons, respectively, for all age groups. (C) Representative TEM images displaying more examples of floating ribbons and ribbon clusters in type I VHCs. (D) Box plot with individual data points from the distance measurements of floating ribbons to the cell membrane. (E) The combination of ribbon counts from the two categories, attached and floating ribbons, shows a remarkable opposing frequency gradient of single and multiple ribbons between the VHC types. (F) Percentages of type I and II VHC ribbons that exhibit a translucent core.

**
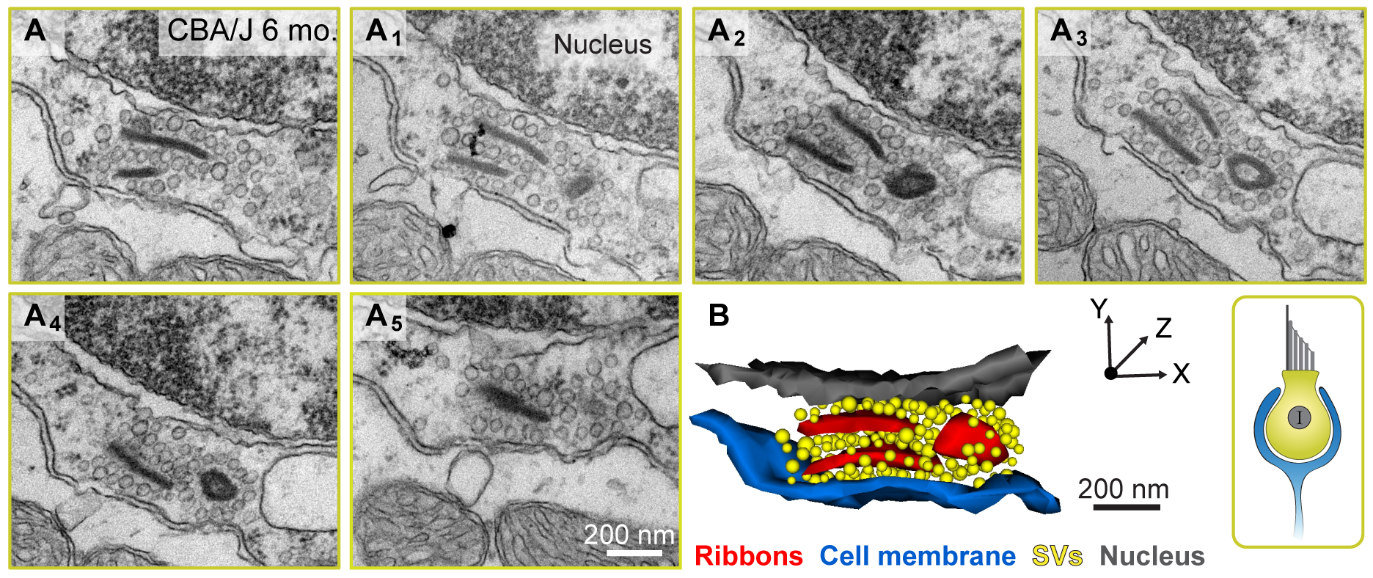
**

**Fig. S2: Floating ribbon clusters are also present in adult CBA/J mice.**

(A-A5) Consecutive ultrathin sections displaying a ribbon cluster of three adjacent floating ribbons and the corresponding 3D model (B).


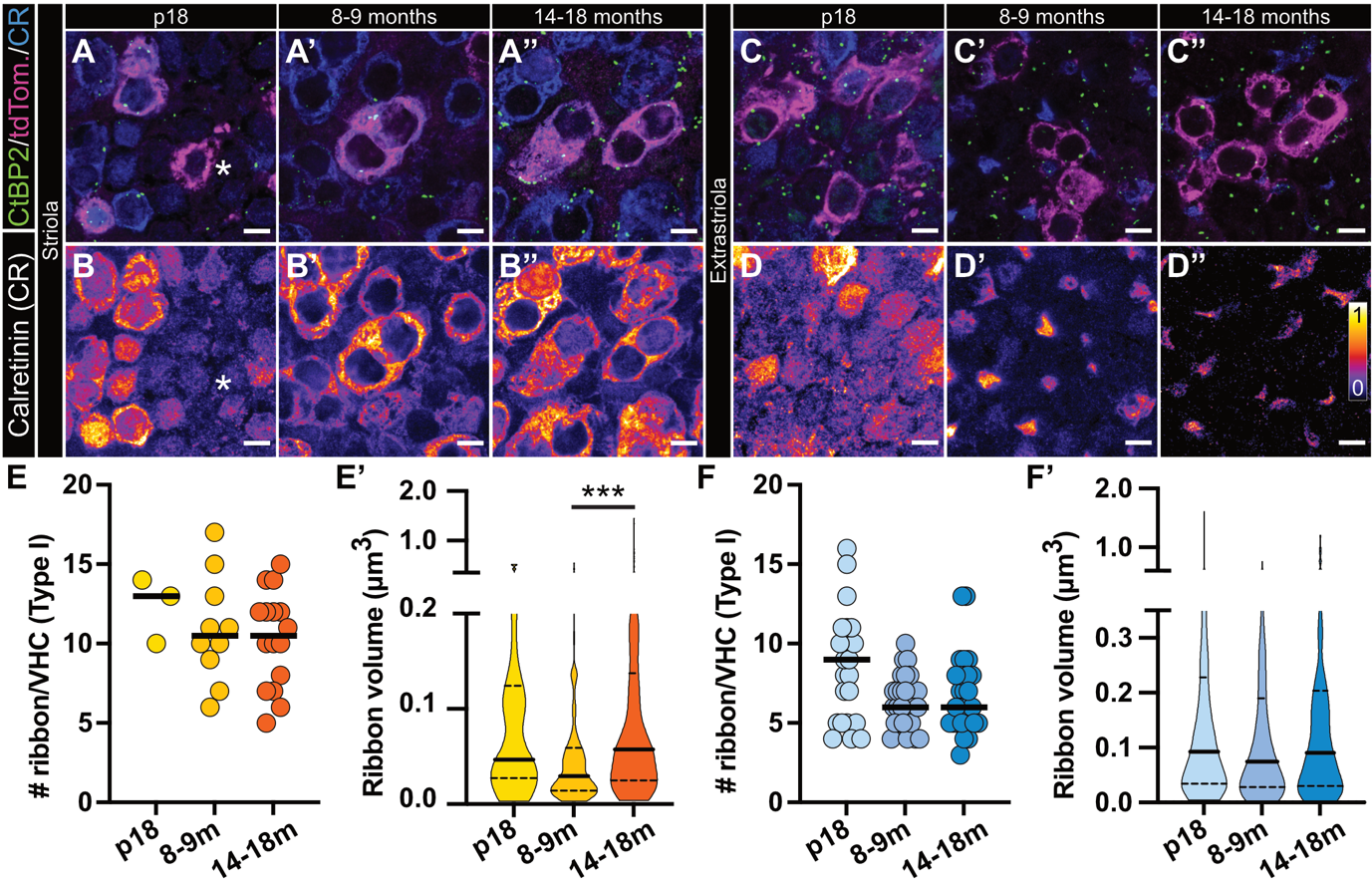


**Fig. S3: Larger synaptic ribbon spots in old type I VHCs in the striola.**

Representative confocal maximum projections of (A-A”) striolar and (C-C”) extrastriolar VHCs of *Ai14-Neurog1-creER^T2^* knock-in mice – that sparsely expresses tdTomato in a random subset of vestibular ganglion neurons – stained for the ribbon marker CtBP2 (green), tdTomato (magenta) and the mobile Ca^2+^-buffer calretinin (CR; blue). (B-B”) and (D-D”) show the individual Calretinin channels for clarity. * indicates a tdTomato-positive, but CR-negative in the adjacent extrastriolar region of (A). (E, F) Plotted are individual cells as well as (E’ and F’) individual ribbon volumes per age group and utricular location. In the striolar region, only VHCs surrounded by calretinin-positive calyces were included in the analysis, whereas in the extrastriolar region only VHCs with CR-negative calyces were analyzed. Ribbon counts in (E and F) consist of data from 3 striolar and 23 extrastriolar type I VHCs from a single P18 animal, while the 8-9-month-group consists of 10 striolar and 28 extrastriolar type I VHCs from three animals and the 1.5-year-group of 16 striolar and 29 extrastriolar type I VHCs from three individuals. Ribbon volumes in (E’ and F’) are displayed with violin plots, where the thick line indicates the median and the thinner dashed lines the inner and outer quartiles. Data were derived from a total of 37, 108 and 174 striolar as well as 196, 180 and 194 ribbons of the respective age groups. ****P*<0.0001 (KW test).


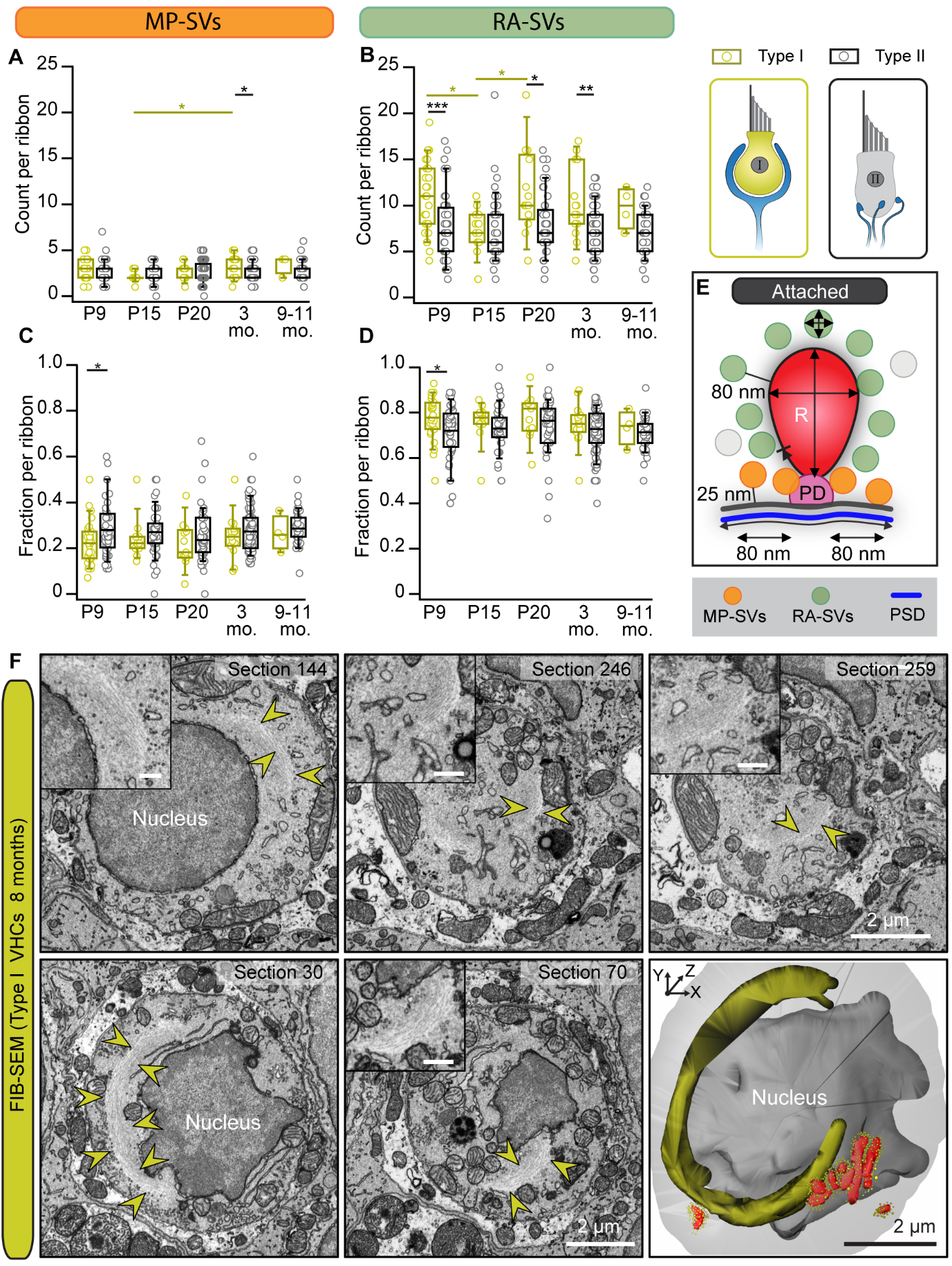


**Fig. S4: Extended strands of microfilaments close to ribbon clusters in mature type I VHCs.**

(A-D) SV count data and their fraction per ribbon for the two different SV pools of attached ribbons. (E) Schemata of the applied quantification criteria for membrane-proximal (MP-) and ribbon-attached synaptic vesicle (RA-SV) pool. (F) Cytoskeletal strands were traced in 3D and are exemplarily highlighted (yellow arrowheads) in individual sections from two different FIB-SEM stacks. In the lower panel, the microfilaments (darker yellow in the 3D model) end close to a ribbon cluster (red objects in the 3D model) at the basal part of the type I VHC. For all insets, the scale bars are 500 nm.

**
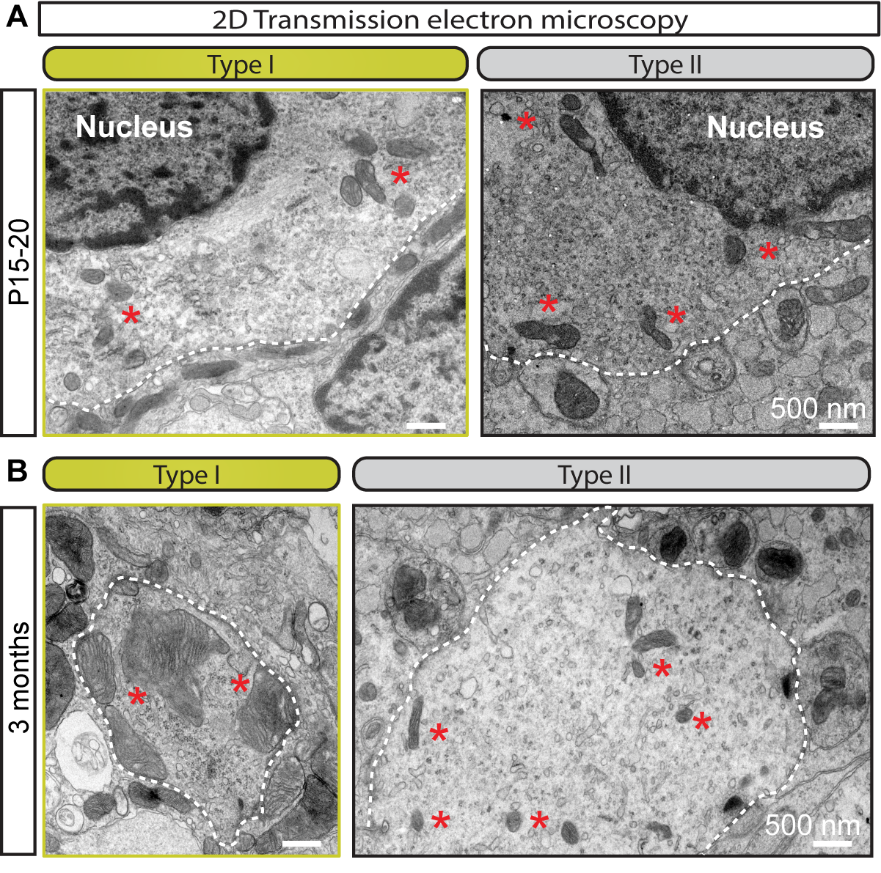
**

**Fig. S5: Subset of mitochondria are enlarged in type I VHCs with advancing age as visualized with 2D electron microscopy.**

Representative electron micrographs highlighting different sized mitochondria (red asterisks) from young ages (A) compared to a mature age (B).

|  | N | n | Ribbon area (nm^2^) x 10^3^ (Att. + Flo.) | Ribbon area (nm^2^) x 10^3^ (Att.) | Ribbon area (nm^2^) x 10^3^  (Flo.) | Ribbon height (nm)  (Att.) | Ribbon width (nm)  (Att.) |
| --- | --- | --- | --- | --- | --- | --- | --- |
| *P9 type I* | 2 | **31** | **12.33 ± 1.27** | **18.70 ± 2.55** | **10.58 ± 1.17** | **194.75 ± 11.33** | **125.12 ± 13.81** |
| *P9 type II* |  | 44 | 14.10 ± 1.31 | 13.86 ± 1.56 | 18.21 ± 2.43 | 157.81 ± 8.50 | 104.48 ± 7.03 |
| *P15 type I* | 2 | **39** | **10.76 ± 0.89** | **13. 27 ± 1.32** | **10.7 ± 1.14** | **179.15 ± 10.54** | **93.21 ± 6.94** |
| *P15 type II* |  | 61 | 13.21 ± 1.25 | 11.51 ± 1.09 | 15.94 ± 3.34 | 145.99 ± 10.90 | 95.09 ± 5.40 |
| *P20 type I* | 2 | **21** | **9.89 ± 0.70** | **15.33 ± 3.40** | **9.50 ± 0.60** | **241.14 ± 22.56** | **67.82 ± 23.01** |
| *P20 type II* |  | 50 | 13.16 ± 1.05 | 13.32 ± 1.23 | 12.13 ± 2.04 | 162.29 ± 6.44 | 116.22 ± 7.22 |
| *3 mo. type I* | 2 | **19** | **15.45 ± 0.97** | **16.58 ± 5.87** | **15.19 ± 0.82** | **181.16 ± 18.03** | **134.33 ± 16.86** |
| *3 mo. type II* |  | 107 | 12.62 ± 0.61 | 12.49 ± 0.68 | 13.06 ± 1.32 | 149.02 ± 3.62 | 103.48 ± 4.13 |
| *9-11 mo.*  *type I* | 2 | **7** | **12.33 ± 0.60** | **17.57 ± 3.45** | **12.30 ± 0.60** | **161.70 ± 25.53** | **97.24 ± 31.00** |
| *9-11 mo.*  *type II* |  | 43 | 13.60 ± 1.67 | 13.63 ± 1.84 | 12.00 ± 4.81 | 148.48 ± 8.90 | 118.58 ± 7.79 |
| *P-values* |  |  | ****** 3 mo. type I vs.  P15 type I  P20 type I  9-11 mo. type I  3 mo. type II  ****** P20 type I vs.  type II  **P* = 0.045  9-11 mo. type I vs. type II  **P* = 0.013 P9 type I vs. P20 type I | **P* = 0.035  P9 type I vs. type II  ****P* = 0.0008  3 mo. type I vs. type II | ***P* = 0.009  P15 type I vs. type II  **P* = 0.011  3 mo. type I vs. type II  **** 3 mo. type I vs.  P15 type I  P20 type I  9-11 mo. type I  ***P* = 0.003  3 mo. type I vs. P9 type I | **** P20 type I vs. type II  ***P* = 0.010  3 mo. type I vs. type II  **P* = 0.027  P15 type I vs. type II  **P* = 0.043  P20 type I vs. P15 type I | **P* = 0.018  P20 type I vs. type II  **P* = 0.028  P9 type I vs. P20 type I |

**Table S1: Ribbon size measurement data from TEM.**

Attached (Att.) and floating (Flo.) ribbon size values from type I and type II VHCs (Supplementary data for Fig. 1 and Fig. S1). Data are presented as median ± SEM. *P*-values are calculated by the two-tailed unpaired Student’s t-test for normally distributed data with equal variances. The unpaired two-tailed Mann-Whitney-Wilcoxon test was applied for not normally distributed data and/or when variances were unequal between two samples. For multiple comparisons, the one-way ANOVA with post-hoc Tukey’s test was used for normally distributed data or in case of non-normally distributed data the KW test with multiple comparison correction was utilized. Significant results are highlighted with **P* < 0.05, ***P* < 0.01, ****P* < 0.001, *****P* < 0.0001. N = animal count, n = ribbon count.

|  | N | n | Ribbon shape in % (absolute number)  round round flat wedge droplet oval elongated | | | | | |
| --- | --- | --- | --- | --- | --- | --- | --- | --- |
| *P9 type I* | 2 | **86** | **17.44**  **(15)** | **8.14**  **(7)** | **13.95**  **(12)** | **10.47**  **(9)** | **22.09**  **(19)** | **27.91**  **(24)** |
| *P9 type II* |  | 63 | 22.22  (14) | 7.94  (5) | 11.11  (7) | 28.57  (18) | 23.81  (15) | 6.35  (4) |
| *P15 type I* | 2 | **125** | **17.60**  **(22)** | **3.20**  **(4)** | **10.40**  **(13)** | **7.20**  **(9)** | **24.80**  **(31)** | **36.80**  **(46)** |
| *P15 type II* |  | 85 | 15.29  (13) | 11.76  (10) | 4.71  (4) | 20.00  (17) | 32.94  (28) | 15.29  (13) |
| *P20 type I* | 2 | **164** | **12.80**  **(21)** | **1.22**  **(2)** | **7.32**  **(12)** | **3.05**  **(5)** | **17.68**  **(29)** | **57.93**  **(95)** |
| *P20 type II* |  | 69 | 26.09  (18) | 7.25  (5) | 5.80  (4) | 23.19  (16) | 27.54  (19) | 10.14  (7) |
| *3 mo. type I* | 2 | **207** | **14.98**  **(31)** | **3.86**  **(8)** | **4.83**  **(10)** | **5.31**  **(11)** | **24.64**  **(51)** | **46.38**  **(96)** |
| *3 mo. type II* |  | 147 | 24.49  (36) | 14.29  (21) | 7.48  (11) | 9.52  (14) | 24.49  (36) | 19.73  (29) |
| *9-11 mo. type I* | 2 | **162** | **22.84**  **(37)** | **2.47**  **(4)** | **2.47**  **(4)** | **4.32**  **(7)** | **23.46**  **(38)** | **44.44**  **(72)** |
| *9-11 mo. type II* |  | 51 | 15.69  (8) | 21.57  (11) | 13.73  (7) | 15.69  (8) | 27.45  (14) | 5.88  (3) |

**Table S2: Evaluation of ribbon shapes observed by random section TEM.**

Percentages of ribbon shapes at type I and type II VHCs. Absolute numbers are given in brackets. N = animal count, n = count of membrane-attached and floating ribbons (Supplementary data for Fig. 1).

|  | N | n | Ribbon count in %  Attached Floating | | Ribbon count in %  Single Multiple | | Ribbon count per cluster in % (absolute cluster number)  2 3 4 5 6 7 8 9 | | | | | | | |
| --- | --- | --- | --- | --- | --- | --- | --- | --- | --- | --- | --- | --- | --- | --- |
| *P9 type I* | 2 | **86** | **36.05**  **(31)** | **63.95**  **(55)** | **38.37**  **(33)** | **61.63**  **(53)** | **40.00**  **(6)** | **33.33**  **(5)** | **13.33**  **(2)** | **6.67**  **(1)** | **-** | **6.67**  **(1)** | **-** | **-** |
| *P9 type II* |  | 63 | 69.84  (44) | 30.16  (19) | 57.14  (36) | 42.86  (27) | 80.00  (4) | 20.00  (1) | - | - | - | - | - | - |
| *P15 type I* | 2 | **128** | **30.47**  **(39)** | **69.53**  **(89)** | **24.22**  **(31)** | **75.78**  **(97)** | **40.00**  **(10)** | **16.00**  **(4)** | **20.00**  **(5)** | **20.00**  **(5)** | **-** | **-** | **4.00**  **(1)** | **-** |
| *P15 type II* |  | 86 | 70.93  (61) | 29.07  (25) | 52.33  (45) | 47.67  (41) | 44.44  (4) | 33.33  (3) | 22.22  (2) | - | - | - | - | - |
| *P20 type I* | 2 | **165** | **12.73**  **(21)** | **87.27**  **(144)** | **15.76**  **(26)** | **84.24**  **(139)** | **34.15**  **(14)** | **29.27**  **(12)** | **21.95**  **(9)** | **4.88**  **(2)** | **7.32**  **(3)** | **2.44**  **(1)** | **-** | **-** |
| *P20 type II* |  | 72 | 69.44  (50) | 30.56  (22) | 83.33  (60) | 16.67  (12) | 100.00  (4) | - | - | - | - | - | - | - |
| *3 mo. type I* | 2 | **219** | **8.68**  **(19)** | **91.32**  **(200)** | **15.53**  **(34)** | **84.47**  **(185)** | **33.93**  **(19)** | **30.36**  **(17)** | **23.21**  **(13)** | **1.79**  **(1)** | **8.93**  **(5)** | **-** | **-** | **1.79**  **(1)** |
| *3 mo. type II* |  | 151 | 70.86 (107) | 29.14  (44) | 82.78  (125) | 17.22  (26) | 77.78  (7) | 22.22  (2) | - | - | - | - | - | - |
| *9-11 mo.*  *type I* | 2 | **169** | **4.14**  **(7)** | **95.86**  **(162)** | **5.92**  **(10)** | **94.08**  **(159)** | **16.22**  **(6)** | **21.62**  **(8)** | **24.32**  **(9)** | **10.81**  **(4)** | **16.22**  **(6)** | **8.11**  **(3)** | **2.70**  **(1)** | **-** |
| *9-11 mo.*  *type II* |  | 53 | 81.13  (43) | 18.87  (10) | 71.70  (38) | 28.30  (15) | 50.00  (1) | 50.00  (1) | - | - | - | - | - | - |

**Table S3: TEM analysis for ribbon counts.**

Percentages of ribbon numbers at type I and type II VHCs. Absolute numbers are given in brackets. N = animal count, n = count of membrane-attached and floating ribbons (Supplementary data for Fig. 2 and Fig. S1).

|  | N | n | Ribbon count in %  attached floating | | Ribbon count in %  single multiple | | Ribbon count per synapse in % (absolute numbers)  1 2 3 4 5 8 9 12 14 | | | | | | | | |
| --- | --- | --- | --- | --- | --- | --- | --- | --- | --- | --- | --- | --- | --- | --- | --- |
| *P15 type I* | 2 | **37** | **51.35**  **(19)** | **48.65**  **(18)** | **29.73**  **(11)** | **70.27**  **(26)** | **64.71**  **(11)** | **17.65**  **(3)** | **5.88**  **(1)** | **-** | **5.88**  **(1)** | **-** | **-** | **5.88**  **(1)** | **-** |
| *P15 type II* | 1 | 26 | 96.15  (25) | 3.85  (1) | 15.38  (4) | 84.62  (22) | 45.45 (5) | 27.27  (3) | 9.09  (1) | 9.09  (1) | - | 9.09  (1) | - | - | - |
| *8 mo. type I* | 2 | **53** | **13.21**  **(7)** | **86.79**  **(46)** | **18.87**  **(10)** | **81.13**  **(43)** | **62.50**  **(10)** | **6.25**  **(1)** | **-** | **-** | **12.50**  **(2)** | **6.25**  **(1)** | **6.25**  **(1)** | **-** | **6.25**  **(1)** |
| *8 mo. type II* |  | 55 | 94.55  (52) | 5.45  (3) | 54.55  (30) | 45.45  (25) | 78.05  (32) | 12.20  (5) | 7.32  (3) | 2.44  (1) | - | - | - | - | - |

**Table S4: FIB-SEM data on ribbon count quantifications.**

Percentages of ribbon numbers at type I and type II VHCs. Absolute numbers are given in brackets. N = animal count, n = total ribbon count (Supplementary data for Fig. 4). P15 type I = 3 VHCs; P15 type II = 1 VHC; 8 mo. type I = 4 VHCs; 8 mo. type II = 2 VHCs.

|  | *P15 type I*  (utricle 1, VHC 1) | *P15 type I*  (utricle 2, VHC 1) | *P15 type I*  (utricle 2, VHC 2) | *P15 type II*  (utricle 1, VHC 1) | *8 mo. type I (utricle 1, VHC 1)* | *8 mo. type I*  (utricle 1, VHC 2) | *8 mo. type I*  (utricle 2, VHC 1) | *8 mo. type I*  (utricle 2, VHC 2) | *8 mo. type II (utricle 1, VHC 1)* | *8 mo. type II (utricle 2, VHC 1)* |
| --- | --- | --- | --- | --- | --- | --- | --- | --- | --- | --- |
| **Ribbons (total)** | **6** | **20** | **11** | **26** | **18** | **10** | **15** | **10** | **31** | **24** |
| attached | 5 | 8 | 6 | 25 | 0 | 3 | 3 | 1 | 28 | 24 |
| floating | 1 | 12 | 5 | 1 | 18 | 7 | 12 | 9 | 3 | 0 |
| **Single ribbons:** | | | | | | | | | | |
| **Single Ribbons (total)** | **4** | **3** | **4** | **4** | **2** | **5** | **2** | **1** | **8** | **22** |
| attached | 3 | 3 | 3 | 4 | 0 | 3 | 2 | 1 | 8 | 22 |
| floating | 1 | 0 | 1 | 0 | 2 | 2 | 0 | 0 | 0 | 0 |
| **Ribbons in clusters:** | | | | | | | | | | |
| **Ribbons in clusters (total)** | **2** | **17** | **7** | **22** | **16** | **5** | **13** | **9** | **23** | **2** |
| attached | 2 | 5 | 3 | 21 | 0 | 0 | 1 | 0 | 20 | 2 |
| floating | 0 | 12 | 4 | 1 | 16 | 5 | 12 | 9 | 3 | 0 |
| **Cluster counts:** | | | | | | | | | | |
| **Cluster count (total)** | **1** | **3** | **2** | **9** | **2** | **1** | **2** | **1** | **9** | **1** |
| with att. ribbons only | 1 | 1 | 1 | 8 | 0 | 0 | 0 | 0 | 8 | 1 |
| with flo. ribbons only | 0 | 0 | 0 | 0 | 2 | 1 | 1 | 1 | 1 | 0 |
| with att. and flo. ribbons | 0 | 2 | 1 | 1 | 0 | 0 | 1 | 0 | 0 | 0 |
| **Ribbons with translucent core:** | | | | | | | | | | |
| Translucent core | 3 | 0 | 0 | 0 | 1 | 1 | 2 | 5 | 1 | 0 |

**Table S5: Counts of different ribbon and cluster categories from FIB-SEM.**

Absolute ribbon counts per VHC (Supplementary data for Fig. 4). Att. = attached, flo. = floating. P15 type I = 3 VHCs, N = 2 animals; P15 type II = 1 VHC,

N = 1 animal; 8 mo. type I = 4 VHCs, N = 2 animals; 8 mo. type II = 2 VHCs, N = 2 animals.

|  | N | n | Individual ribbon volume (nm^3^) x 10^6^ | Total ribbon volume per contact/cluster (nm^3^) x 10^6^ | Total SV count per contact/cluster |
| --- | --- | --- | --- | --- | --- |
| *P15 type I* | 2 | **37** | **0.87 ± 0.86** | **3.39 ± 2.75** | **47 ± 24.65** |
| *P15 type II* | 1 | 26 | 0.43 ± 0.05 | 0.99 ± 0.44 | 37 ± 13.06 |
| *8 mo. type I* | 2 | **53** | **3.49 ± 0.79** | **7.12 ± 6.59** | **83 ± 50.89** |
| *8 mo. type II* |  | 55 | 1.40 ± 0.54 | 1.40 ± 1.29 | 28 ± 3.66 |
| *P-values* |  |  | ***P* = 0.003  P15 type I vs. type II  ***P* = 0.004  8 mo. type I vs. type II  *****P* P15 type II vs. 8 mo. type II  **P* = 0.025  P15 type I vs. 8 mo. type I | ***P* = 0.005  8 mo. type I vs. type II | **P* = 0.018  P15 type I vs. 8 mo. type I  *****P* 8 mo. type I vs. type II |

**Table S6: FIB-SEM data on SV count and ribbon size measurements.**

Ribbon size values and SV counts from type I and type II VHCs, respectively (Supplementary data for Fig. 4). Data are presented as median ± SEM.

Significant results are highlighted with **P* < 0.05, ***P* < 0.01, ****P* < 0.001, *****P* < 0.0001. N = animal count, n = total ribbon count. P15 type I = 3 VHCs; P15 type II = 1 VHC; 8 mo. type I = 4 VHCs; 8 mo. type II = 2 VHCs.

|  | N | n | SV count per ribbon  Attached Floating MP-SV RA-SV | | | | SV density per ribbon surface (x 10^-3^) Attached | SV density per ribbon surface (x 10^-3^) Floating | Fraction per ribbon MP-SV RA-SV | |
| --- | --- | --- | --- | --- | --- | --- | --- | --- | --- | --- |
| *P9 type I* | 2 | **86** | **13.0 ± 0.74** | **13.0 ± 0.67** | **3.0 ± 0.20** | **11.0 ± 0.70** | **0.66 ± 0.10** | **0.89 ± 0.10** | **0.22 ± 0.02** | **0.78 ± 0.02** |
| *P9 type II* |  | 63 | 9.0 ± 0.64 | 11.5 ± 0.98 | 3.0 ± 0.18 | 7.0 ± 0.57 | 0.69 ± 0.05 | 0.82 ± 0.14 | 0.28 ± 0.02 | 0.72 ± 0.02 |
| *P15 type I* | 2 | **128** | **9.0 ± 0.65** | **12.0 ± 1.53** | **2.0 ± 0.13** | **7.0 ± 0.57** | **0.68 ± 0.10** | **0.76 ± 0.10** | **0.22 ± 0.02** | **0.78 ± 0.02** |
| *P15 type II* |  | 86 | 9.0 ± 0.63 | 12.0 ± 0.44 | 2.0 ± 0.13 | 6.0 ± 0.53 | 0.73 ± 0.05 | 0.73 ± 0.10 | 0.27 ± 0.01 | 0.73 ± 0.01 |
| *P20 type I* | 2 | **165** | **14.0 ± 1.23** | **15.0 ± 0.90** | **3.0 ± 0.23** | **10.0 ± 1.30** | **1.08 ± 0.13** | **0.96 ± 0.00** | **0.18 ± 0.03** | **0.82 ± 0.03** |
| *P20 type II* |  | 72 | 9.0 ± 0.53 | 11.0 ± 0.72 | 2.0 ± 0.16 | 7.0 ± 0.46 | 0.75 ± 0.00 | 0.74 ± 0.18 | 0.24 ± 0.02 | 0.76 ± 0.02 |
| *3 mo. type I* | 2 | **219** | **13.0 ± 1.09** | **16.0 ± 0.76** | **3.0 ± 0.30** | **9.0 ± 1.00** | **0.60 ± 0.10** | **0.56 ± 0.09** | **0.25 ± 0.02** | **0.75 ± 0.02** |
| *3 mo. type II* |  | 151 | 10.0 ± 0.27 | 11.0 ± 0.60 | 3.0 ± 0.09 | 7.0 ± 0.26 | 0.75 ± 0.04 | 0.88 ± 0.08 | 0.27 ± 0.01 | 0.73 ± 0.01 |
| *9-11 mo.*  *type I* | 2 | **169** | **13.0 ± 1.31** | **14.0 ± 1.58** | **4.0 ± 0.50** | **10.0 ± 1.11** | **0.69 ± 0.17** | **0.92 ± 0.15** | **0.26 ± 0.04** | **0.74 ± 0.04** |
| *9-11 mo.*  *type II* |  | 53 | 10.0 ± 0.49 | 9.0 ± 1.13 | 3.0 ± 0.16 | 7.0 ± 0.38 | 0.66 ± 0.04 | 0.84 ± 0.10 | 0.29 ± 0.01 | 0.71 ± 0.01 |
| *P-values* |  |  | ****P* = 0.0005  P9 type I vs. type II  ***P* = 0.004  P20 type I vs. type II  ****P* = 0.0007  3 mo. type I vs. type II  **P* = 0.015  P15 type I vs. P20 type I  **P* = 0.033  P15 type I vs. 3 mo. type I  ***P* = 0.009  P15 type I vs. P9 type I | *****P*  3 mo. type I vs. type II  ***P* = 0.002  P20 type I vs. type II  **P* = 0.046  P15 type I vs. 3 mo. type I | **P* = 0.040  3 mo. type I vs. type II  **P* = 0.016  3 mo. type I vs. P15 type I | ****P* =0.0004  P9 type I vs. type II  ***P* = 0.002  3 mo. type I vs. type II  **P* = 0.021 P20 type I vs. type II  **P* = 0.028 P9 type I vs. P15 type I  **P* = 0.021 P20 type I vs. P15 type I | *n.s.* | **P* = 0.049 P20 type I vs. 3 mo. type I  **P* = 0.021  3 mo. type I vs. type II | **P* = 0.019 P9 type I vs. type II | **P* = 0.019  P9 type I vs. type II |

**Table S7: TEM data on SV counts.**

Number of SVs per ribbon from type I and type II VHCs, respectively (Supplementary data for Fig. 5 and Fig. S4). Data are presented as median ± SEM. Depending on the normality and the equality of variances tests, p-values are calculated by the two-tailed unpaired Student’s t-test or the unpaired two-tailed Mann-Whitney-Wilcoxon test between two samples. For multiple comparisons, the one-way ANOVA with post-hoc Tukey’s test or the KW test with multiple comparison correction was utilized. Non-significant differences are highlighted with *n.s.* and significant results are highlighted with **P* < 0.05, ***P* < 0.01, ****P* < 0.001, *****P* < 0.0001. N = animal count, n = total ribbon count.

|  | N | n | SV diameter (nm)  Attached Floating | | Distance floating ribbon to membrane (nm) |
| --- | --- | --- | --- | --- | --- |
| *P9 type I* | 2 | **86** | **46.33 ± 0.34** | **45.77 ± 0.34** | **237.73 ± 31.81** |
| *P9 type II* |  | 63 | 48.83 ± 0.61 | 46.72 ± 0.60 | 107.61 ± 108.15 |
| *P15 type I* | 2 | **128** | **48.95 ± 0.80** | **44.39 ± 0.48** | **271.18 ± 49.77** |
| *P15 type II* |  | 86 | 50.76 ± 0.57 | 50.82 ± 0.91 | 303.80 ± 46.77 |
| *P20 type I* | 2 | **165** | **46.96 ± 0.95** | **45.68 ± 0.33** | **259.79 ± 21.28** |
| *P20 type II* |  | 72 | 48.92 ± 0.62 | 47.52 ± 0.60 | 97.35 ± 73.68 |
| *3 mo. type I* | 2 | **219** | **45.54 ± 0.40** | **45.69 ± 0.23** | **269.29 ± 19.07** |
| *3 mo. type II* |  | 151 | 51.27 ± 0.27 | 52.98 ± 0.52 | 170.87 ± 30.43 |
| *9-11 mo.*  *type I* | 2 | **169** | **44.32 ± 0.79** | **43.08 ± 0.55** | **266.50 ± 16.57** |
| *9-11 mo.*  *type II* |  | 53 | 49.66 ± 0.45 | 47.93 ± 0.76 | 123.32 ± 74.97 |
| *P-values* |  |  | ****** P9 type I vs. type II; ****** P15 type I vs. type II; ****** P20 type I vs. type II; ****** 3 mo. type I vs. type II; ****** 9-11 mo. type I vs. type II  ****** P20 type I vs. 9-11 mo. type I  ****P* = 0.0002 P15 type I vs. 9-11 mo. type I  ***P* = 0.001 P15 type I vs. P9 type I  ***P* = 0.002 P15 type I vs. 3 mo. type I  ***P* = 0.007 P9 type I vs. 9-11 mo. type I  **P* = 0.013 3 mo. type I vs. 9-11 mo. type  **P* = 0.038 P20 type I vs. 3 mo. type I  ****** 3 mo. and 9-11 mo. type II vs. P9 type II  ****** 9-11 mo. type II vs. P15 type II  ****P* = 0.0006 9-11 mo. type II vs. P20 type II  ***P* = 0.005 3 mo. type II vs. 9-11 mo. type II  ***P* = 0.003 3 mo. type II vs. P15 type II  **P* = 0.015 3 mo. type II vs. P20 type II | ***P*= 0.0078 P9 type I vs. type II; ****** P15 type I vs. type II; ****P*= 0.0002 P20 type I vs. type II; ****** 3 mo. type I vs. type II; ****** 9-11 mo. type I vs. type II  ****** 3 mo. type II vs. P9 type II, P20 type II and 9-11 mo. type II  ****P* = 0.0003 P9 type II vs. P15 type II  ***P* = 0.006 3 mo. type II vs. P15 type II  **P* = 0.020 9-11 mo. type II vs. P15 type II  **P* = 0.029 P15 type II vs. P20 type II | ****P* = 0.0002 P20 type I vs. type II  **P* = 0.036 3 mo. type I vs. type II |

**Table S8: Size and distance measurements of AZ parameters.**

SV size and floating ribbon distance values at type I and type II VHCs (Supplementary data for Fig. 5 and Fig. S1). Data are presented as median ± SEM. Significant results are highlighted with **P* < 0.05, ***P* < 0.01, ****P* < 0.001, *****P* < 0.0001. N = animal count, n = total ribbon count. P-values are calculated by the two-tailed unpaired Student’s t-test or the unpaired two-tailed Mann-Whitney-Wilcoxon test between two samples. For multiple group comparisons, the KW test with multiple test correction was utilized.

|  | N | n | Feeding days | ^15^N/^14^N ratio | Categories | *P-values* |
| --- | --- | --- | --- | --- | --- | --- |
| *Attached ribbons* | 2 | 4 | 14 days | 1.14 ± 0.16 | floating vs heterochromatin | *****P* < 0.0001 |
| *Floating ribbons* |  |  |  | 1.01 ± 0.05 | floating vs euchromatin | ****P* = 0.0003 |
| *Mitochondria* |  |  |  | 0.80 ± 0.02 | floating vs mitochondria | *****P* < 0.0001 |
| *Cytosol* |  |  |  | 1.00 ± 0.02 | floating vs attached | *n.s. P* = 0.741 |
| *Euchromatin* |  |  |  | 0.72 ± 0.02 | floating vs cytosol | *n.s. P* = 0.816 |
| *Heterochromatin* |  |  |  | 0.13 ± 0.03 | cytosol vs heterochromatin | *****P* < 0.0001 |
|  |  |  |  |  | cytosol vs euchromatin | ***P* = 0.002 |
|  |  |  |  |  | cytosol vs mitochondria | *****P* < 0.0001 |
|  |  |  |  |  | cytosol vs attached | *n.s. P* = 0.986 |
|  |  |  |  |  | attached vs heterochromatin | *****P* < 0.0001 |
|  |  |  |  |  | attached vs euchromatin | *n.s. P* = 0.127 |
|  |  |  |  |  | attached vs mitochondria | *n.s. P* = 0.248 |
|  |  |  |  |  | mitochondria vs heterochromatin | *****P* < 0.0001 |
|  |  |  |  |  | mitochondria vs euchromatin | *n.s. P* = 0.869 |
|  |  |  |  |  | euchromatin vs heterochromatin | *****P* < 0.0001 |
| *Attached ribbons* | 2 | 4 | 21 days | 1.25 ± 0.04 | attached vs heterochromatin | *****P* < 0.0001 |
| *Floating ribbons* |  |  |  | 1.19 ± 0.03 | attached vs euchromatin | *****P* < 0.0001 |
| *Mitochondria* |  |  |  | 0.81 ± 0.02 | attached vs mitochondria | ***P* = 0.002 |
| *Cytosol* |  |  |  | 1.01 ± 0.01 | attached vs cytosol | *n.s. P* = 0.624 |
| *Euchromatin* |  |  |  | 0.60 ± 0.04 | attached vs floating | *n.s. P* = 1.000 |
| *Heterochromatin* |  |  |  | 0.11 ± 0.01 | floating vs heterochromatin | *****P* < 0.0001 |
|  |  |  |  |  | floating vs euchromatin | *****P* < 0.0001 |
|  |  |  |  |  | floating vs mitochondria | *****P* < 0.0001 |
|  |  |  |  |  | floating vs cytosol | *n.s. P* = 0.053 |
|  |  |  |  |  | cytosol vs heterochromatin | *****P* < 0.0001 |
|  |  |  |  |  | cytosol vs euchromatin | ****P* = 0.0004 |
|  |  |  |  |  | cytosol vs mitochondria | **P* = 0.031 |
|  |  |  |  |  | mitochondria vs heterochromatin | ***P* = 0.008 |
|  |  |  |  |  | mitochondria vs euchromatin | *n.s. P* = 1.000 |
|  |  |  |  |  | euchromatin vs heterochromatin | *n.s. P* = 0.453 |

**Table S9: NanoSIMS analysis data from animals fed with ^15^N.**

Data are presented as median ± SEM. Significant results are highlighted with **P* < 0.05, ***P* < 0.01, ****P* < 0.001, *****P* < 0.0001. N = animal count, n = utricle count. P-values are calculated by the ANOVA test followed by a post-hoc Tukey test for the 14 days feeding data, while for the 21 days feeding data the KW test with multiple test correction was utilized.
